# Examination of effects of indoor fires on building structures and people

**DOI:** 10.1016/j.heliyon.2022.e12720

**Published:** 2022-12-30

**Authors:** Rajmund Kuti, Géza Zólyomi, Gabriella László, Csaba Hajdu, László Környei, Flóra Hajdu

**Affiliations:** a Department of Automation and Mechatronics, Faculty of Mechanical Engineering, Informatics and Electrical Engineering, Széchenyi István University, Győr, Hungary; b Directorate for Disaster Management of Heves County, Gyöngyös Disaster Department, Hungary; c Doctoral School of Multidisciplinary Engineering Sciences, Széchenyi István University, Győr, Hungary; d Department of Mathematics, Faculty of Mechanical Engineering, Informatics and Electrical Engineering, Széchenyi István University, Győr, Hungary; e Department of Machine Design, Faculty of Mechanical Engineering, Informatics and Electrical Engineering, Széchenyi István University, Győr, Hungary

**Keywords:** Indoor fire, Building structure, Fire load, Fire experiment, Numerical simulation

## Abstract

The scientific study of the harmful effects of indoor fires on building structures and on the environment is a top issue today. Indoor fires frequently occur all over the world. The goal of our research is to examine the effects of an average room fire on the survival possibility of a trapped person and on the building structure, taking into account features of the Eastern European architecture. First, a computational fluid dynamics (CFD) simulation was performed to examine the change of temperature, oxygen, and carbon monoxide concentration in a selected room in a vacant building used for military training. Based on the results, a 1:1 scale fire experiment was carried out with the parameters used in the simulation. The experiment was repeated once with the same settings. It was observed that without the intervention of firefighters, the temperature in the experiment could have rapidly reached 400 °C, as suggested by the simulation, which could have caused structural damage to the building. Furthermore, after 3 min the carbon monoxide concentration reached 400 ppm in both experiments and the simulation, which is a harmful level to people trapped inside the room. Also, in the experiment there was sufficient oxygen at the ground level with what people can survive 3 min.

## 1 Introduction

Most enclosed fires occur in residential buildings, especially in bedrooms or living rooms. According to statistics, the third most common cause of death in Hungary is burning, and suffocation caused by fire [1]. One of the reasons is ageing population. Elderly people are at an increased risk of setting fire while have a disadvantage at escaping [[2], [3], [4], [5], [6]] The most common sources of fire in residential buildings are careless accidents in the kitchen, outdated electrical systems, failures of electrical or heating devices, smoking, use of candles, leakage of gas, improper usage and storage of combustible liquids, and arson [1–5].

During fires in closed spaces, the heat radiation generated by the hot combustion products collected under the ceiling promotes the spread of the fire and the intensive rising of temperature. These combustion products endanger entrapped people and can damage the building structures. A decrease in oxygen level results in even more intense smoke generation. Therefore, the escape routes become saturated with smoke in a short time, which significantly slows the escape and makes it more difficult for firefighters to intervene [7].

In the case of fires in living spaces, high temperatures may cause damage to the building structure. Human survivability is determined by the concentration of toxic gases such as carbon monoxide (CO) [8]. Inhalation of air containing 0.4 vol% of carbon monoxide for 5 min is fatal [9]. Fire-induced pressure rise can also lead to structural damage and can hinder the safe evacuation of the occupants [10].

There is a broad literature on the ways of fire spread [11,12], how the different openings affect the pressure caused by fire [13], or the heat flux [14] and with the effect of ventilation on fire dynamics [15]. In Ref. [12] for instance, fire spread in different rooms of a residential building is examined and compared with a 1:1 scale test. However, there is little information on the possibilities of the escape of a trapped person or on the effect of the fire on building materials.

Simulations can be used for analyzing the behavior of building materials and structures in fire effectively. In Ref. [16] concrete panels filled with insulation are examined in fire. Xu Dong and others measured the temperature of the upper air layer in a closed room and also the temperature of the structure (steel beams and CFT columns) in different positions by a simulation [17]. But examinations of concrete structures during fire [18] and the density of insulation in combined fireproof cladding [19] can be also found.

Sufficient knowledge and assessment of fire behavior can be obtained by 1:1 scale tests and computer simulations simultaneously according to the results of studies in Ref. [20]. In that paper, combustible materials were investigated in different types of residential buildings. Despite these only a few publications can be found about fire simulations in domestic literature. Three fire cases were investigated with CFD models in FDS software. One of them occurred in a residential container [21], and the rest of them were fires in panel buildings [22,23]. Literature sources examining the survival of a person trapped in a room during an actual fire experiment have not been found. Therefore, an important task is to study the effects of heat damaging building structures and people trapped inside and the harmful components of the generated smoke.

Most enclosed fires occur in residential buildings, so in this paper fire in a selected average size room was examined first using the Fire Dynamic Simulator (FDS) simulation software. Afterwards the simulation was replicated with a 1:1 scale fire experiment two times. The temperature, the carbon monoxide (CO) concentration, and the oxygen (O_2_) concentration were measured with sensors in both cases. To prevent actual damage in building structure, the experiment was interrupted and firefighters intervened, when the measured temperature reached 300 °C.

## 2 Material and methods

**Fig. 1:**
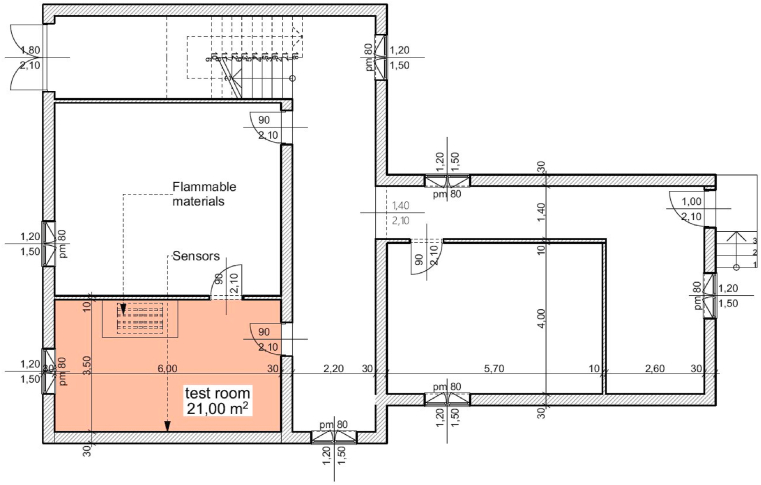
Floor plan of the building of the experiment.

The site of our experiments was at a mixed-use building, formerly military baracks, currently used only for military training, There are no utilities (gas, water, electricity) in it, only military training is held there. The building has two floors, with front and rear exits, several rooms, and a design like residential buildings. The chosen building was built in the 1970’s. The building materials (brick masonry, E-beam slab with linings, smoothed concrete subfloor, standard doors, and windows) and technology were used in many residential buildings built at that time, which are still in use today. The main and partition walls of the building were made of brick, the ceiling was made using reinforced concrete beams and concrete lining bodies, and the floor was smoothed concrete. The floor plan is illustrated in Fig. 1.

**Fig. 2:**
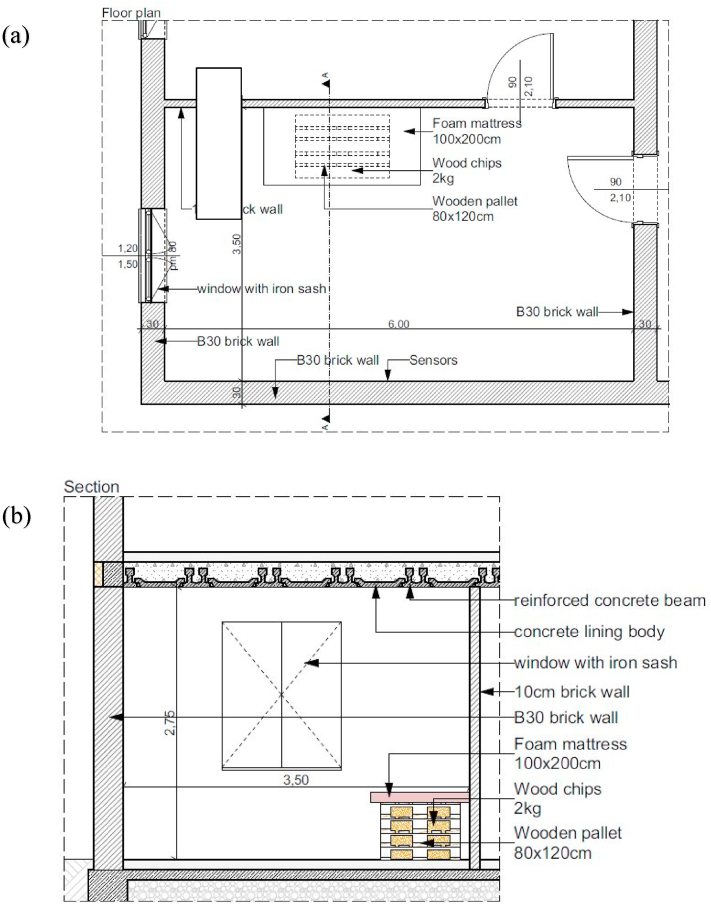
The experiment room: (a): Floor plan, (b): Cross section.

The internal height of the rooms in Hungary is 2.75 m, in general. The experimental room is 3.5 m wide and 6 m long as in an average bedroom. The room has a 1.2 m wide, 1.5 m high window and two 0.9 m wide, 2.1 m high doors. As the current purpose of the building is military training, the doors and windows have an iron sash and frame structure, and the windows are completely covered with iron plates. The doors and windows are closed tightly, performing their function during the experiments. However, there was a 2 cm gap under each door. Fire spread was examined in this room with numerical simulation and 1:1 scale fire experiments. The same amount of combustible material was used for the unit of fire in each case, which consisted of 4 wooden pallets, 2 kg of wood shaving, and one foam mattress (Fig. 2). Pallets were selected as they are widely used for fire experiments [[24], [25], [26], [27]]. A foam mattress was selected as it typically burns in a compartment fire [28] Wood shaving was used to start the fire [29]. The flammable materials were piled up next to the wall because in reality the bed is also next to the wall and many fires start in bed from smoking. It would have been unnecessary to pile up more combustible materials because the temperature generated by the fire was not allowed to exceed 300 °C, which we quickly reached even with this quantity.

### 2.1 Numerical simulation

For numerical simulation, the Fire Dynamic Simulator (FDS) software developed by NIST was used. The latest version is available in 2022. “FDS is a computational fluid dynamics (CFD) model of fire-driven fluid flow. FDS solves numerically a form of the Navier-Stokes equations appropriate for low-speed, thermally-driven flow with an emphasis on smoke and heat transport from fires.” [30]. The major features of the model include low Mach large Eddy simulations, a simple immersed boundary method for treatment of flow obstructions, a generalized lumped species method, and many other concepts. “The most basic description of the chemistry of fire is a reaction of a hydrocarbon fuel with oxygen that produces carbon dioxide and water vapor.” [31] FDS includes source terms and boundary conditions that describe the turbulent combustion of gaseous fuel and oxygen, the transport of thermal radiation through hot, soot-laden gases, and the thermal decomposition of real materials. It uses a combustion model based on the mixing-limited, infinitely fast reaction of lumped species (mixture of species). “The heat release rate per unit volume is defined by summing the lumped species mass production rates times their respective heats of formation (Eq. (1)):”

(1) $${\dot{q}}^{\prime \prime \prime }=-\sum_{\alpha }{\dot{m}}_{\alpha }^{\prime \prime \prime }\Delta h_{f,\alpha }$$

Variables such as lumped species mass fractions, velocity vector, and temperature are calculated in each step with an explicit second-order predictor/corrector scheme. More details about the models can be found in Ref. [31].

FDS is a reliable and effective numerical tool for fire spread simulation [32]. FDS can be used for indoor fire simulation [11,33,34] outdoor fire simulation [35,36] and evacuation simulation [37,38]. FDS offers two types of models for fire-spread simulation. In the first case, fire can be defined by its HRRPUA (heat release rate per unit area) value. In the other case, fire spread and combustion depend entirely on the material properties [30]. The material-based model was chosen because the combustion characteristics of all combustible materials used in the experiments were known. Therefore, the boundary conditions required for computer simulation could be given more accurately.

The input of FDS is a single ASCII text file. In the text file, the simulation space is defined by the size of the mesh. Then the reaction gas and the material properties (Table 1) and the surface of the obstruction (Table 2) are given. The obstructions (pallet, wood shaving, and foam mattress) are specified based on the given surfaces. Then the ignition particles and their properties and the gap under the doors are given. Next, the output variables, which are temperature, CO concentration, and O_2_ concentration are specified. The output is a CSV file, which includes these output variables.

**Table 1:** Material properties used in the simulation.

Material name	Mass (kg)	Density (kg/m^3^)	Specific heat (kJ/kg/K)	Conduc-tivity (W/m/K)	Reference temperature (°C)	Heat of reaction (kJ/kg)	Heat of combustion (kJ/kg)
Wood (pallet)	80	600	1.76	0.12	260	6500	16,750
Wood (shaving)	2	600	1.76	0.12	260	6500	16,750
Fabric	5	100.0	1.34	0.012	340	2800	20,930
Foam	20	40.0	2.7	0.05	420	739	46,470
Brick		1600	0.84	0.69			
Concrete		2300	1.05	1.45			
Steel		7850	0.6	45

**Table 2:** Surface properties used during the simulation.

Name	Color (see Fig. 2)	Materials	Material mass fraction	Thickness
Foam mattress	Green	Fabric; Foam	0.2; 0.8	0.002; 0.1
Pallet	Purple	Wood (pallet)		0.02
Wood chip	Blue	Wood (chip)		0.001
Wall	Light grey	Brick		0.1
Floor	Dark grey	Concrete		0.1
Slab	Yellow	Concrete, steel	0.96; 0.06	0.1; 0.1

The materials used for the experiment and their combustion characteristics are listed in Table 1. The material properties were based on given by scientific literature, the FDS manual, and material tables [30,[39], [40], [41], [42], [43]].

The surfaces for the obstacles are summarized in Table 2.

The fire was started with 1000 °C sparks. The mesh was 5 × 5 × 2 cm to include the 2 cm gap at the doors. According to Ref. [11], the mesh size is adequate in the case of a room of this size. To get the optimal mesh size the heat release rate was estimated, which was 3500 kW [44,45].

The mesh size can be calculated as follows using a fine mesh [30,46] (Eq. (2)):

(2) $$\frac{D^{*}}{16}=dx$$

where $D^{*}$ is a characteristic fire diameter and can be calculated with Eq. (3).

(3) $$D^{*}={\left(\frac{\dot{Q}}{\rho c_{p}T_{amb}\sqrt{g}}\right)}^{\frac{2}{5}}$$

where $\dot{Q}=3500\;kW$ is the heat release rate, $\rho =1.204\frac{kg}{m^{3}}$ is the density of the air, $c_{p}=1.005\frac{kJ}{kg∙K}$ is the specific heat, $T_{amb}=293\;K$ is the ambient temperature, and $g=9.81\frac{m}{s^{2}}$ is the gravity constant. Using [46] calculator the fine mesh size is 10 cm. Based on the literature this size is adequate in case of room fires [47]. The selected mesh size is smaller than recommended for fine mesh consequently no mesh sensitivity study was carried out.

For the reaction, a simple chemistry model was used. The reaction gas was a mixture of wood and polyurethane based on their mass fraction [48,49]:

Sootyield=0.06;COyield=0.06;C=3;H=5;O=2;N=0.015.

**Fig. 3:**
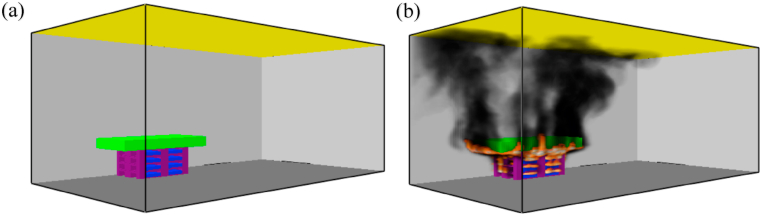
Simulation setup: (a): Before ignition, (b): After ignition.

The temperature change, the oxygen (O_2_) concentration, and the carbon monoxide (CO) concentration were examined at 0.3 and 2.6 m height. The simulation model setup is shown in Fig. 3. The wood shaving was placed in the empty space between the pallets.

#### 2.1.1 1:1 scale fire experiment

The experiments were carried out in 2019 before the COVID-19 virus, in the season of autumn. The ambient temperature was 15 °C in the case of the first and 17 °C in the case of the second experiment. In order to preserve the condition of the building, the operator of the facility had requested that the internal temperature should not rise above 300 °C during the fire experiments. Professional fire brigade units were involved in satisfying this requirement.

The fire was started with a diesel ignition rod. The same firefighter carried out the preparation of the unit of fire, fire ignition, recording of the results, and fire extinguishing according to the same methodology. During the experiments, the temperature change (at 0.3 and 2.6 m height) and the volume percentage change of O_2_ and CO were obtained as a function of time. The sensors were placed 15 cm below the ceiling and 30 cm above the floor. The upper-temperature sensor was not mounted directly on the ceiling so that the temperature could be monitored in the specified area. The lower sensors were placed at 30 cm upwards from the ground so that the survival chance of a person lying in the room could be examined during the time interval of the experiments. It is known that during fires, especially when different materials like wood, plastic, etc. burn a variety of toxic combustion products are produced, but we only had O_2_ and CO meters available for on-site measurements.

**Fig. 4:**
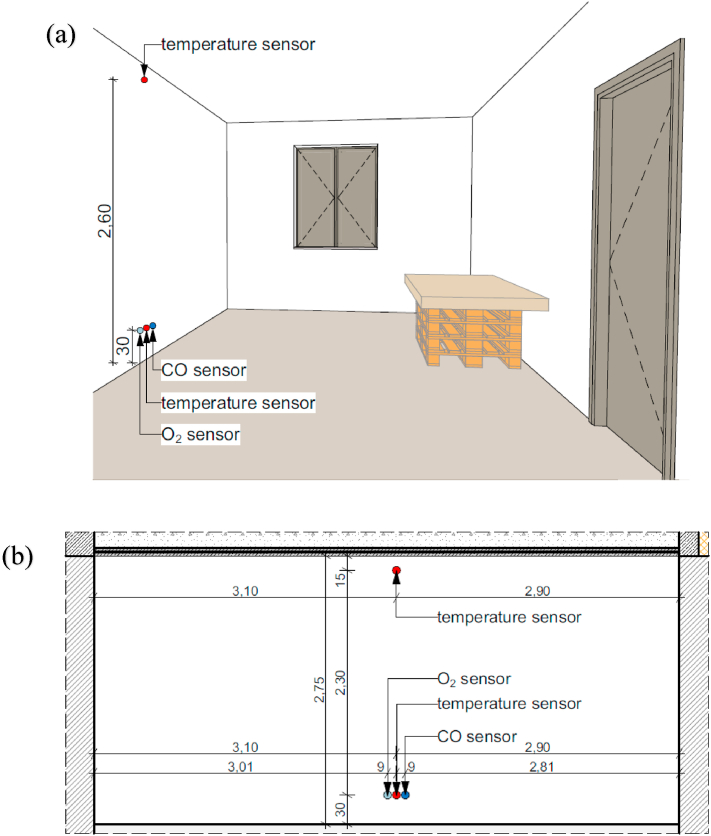
The experimental site: (a): Setup of the experimental site, (b): Placement of the sensors.

**Fig. 5:**
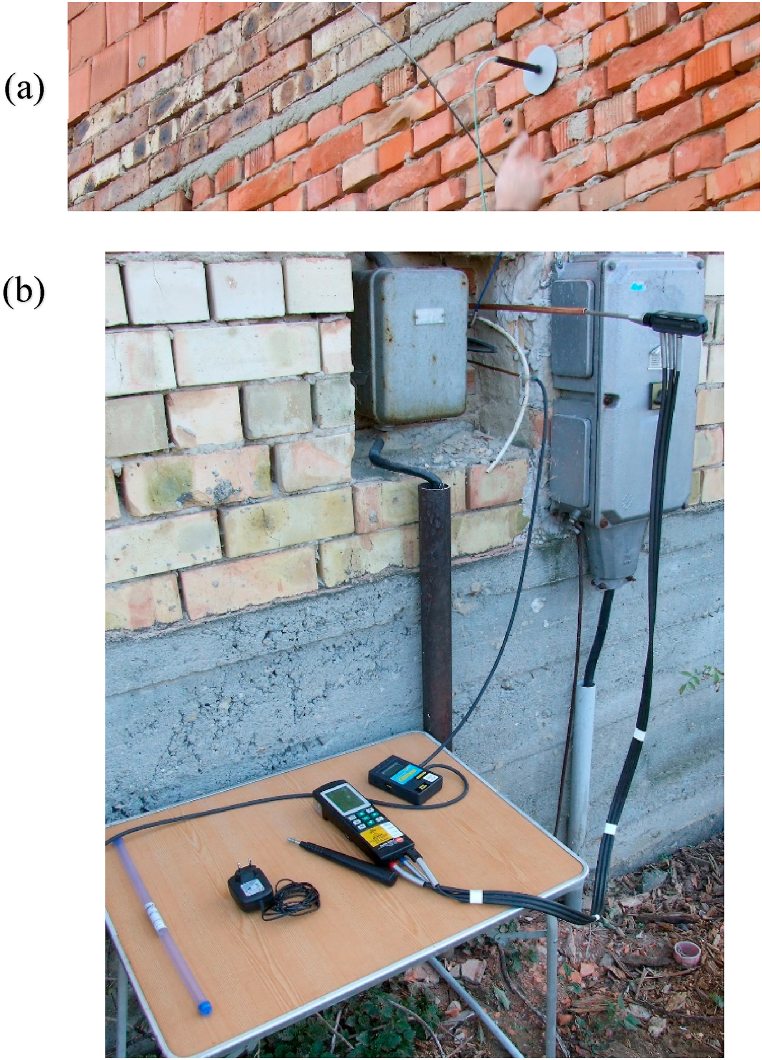
Photos of the sensors: (a): Tepcel Thermometer, (b): RS-232 Thermolog and Testo 325-1 M analyzer.

The equipment in the room and the placement of the sensors are shown in Figs. 4 and 5.

A Tecpel Thermometer and an RS-232 Thermolog instrument were used for temperature measurement. Testo 325-1 M analyzer was used for combustion product evaluation. The time was measured with certified Agat-type stopwatches. During both experiments, the unit of fire was extinguished using a “1 C” water jet, which was put on standby by the firefighters on site before the start of the experiments.

## 3 Calculation

Before starting the simulation, the first step was to calculate the temporary fire load [50] using Eq. (4), taking into account the data in Table 1:

(4) $$p_{n}=\frac{\sum_{j=1}^{n}M_{i}H_{i}}{S}$$

where,

• p_n_ temporary fire load (MJ/m^2^)
• M_i_ the mass of the n-th combustible material (kg)
• H_i_ the calorific value of the n-th combustible material (MJ/kg)
• S the floor area of building (m^2^)
• j the number of materials included in temporary fire load

The following value was calculated for the fire load (Eq. (5)):

(5) $$p_{n}=\frac{\sum_{j=1}^{n}M_{i}H_{i}}{S}=\frac{2407,55}{21}=114,64\;MJ/M^{2}$$

In this case, building structures do not contain combustible material. Therefore, a constant fire load does not need to be calculated. Summarizing the temporary and permanent fire loads, we get the calculated fire load, which in our case is the same as the temporary fire load.

The temporary fire load had to be calculated to have information on the heat effect damaging the building structures. Thus, it can be stated that the fire load generated during the combustion of the materials used in the experiments does not exceed the normative fire load specified during the construction of the building, which is 400 MJ/m^2^ [51].

## 4 Results and discussion

### 4.1 Numerical simulation

**Fig. 6:**
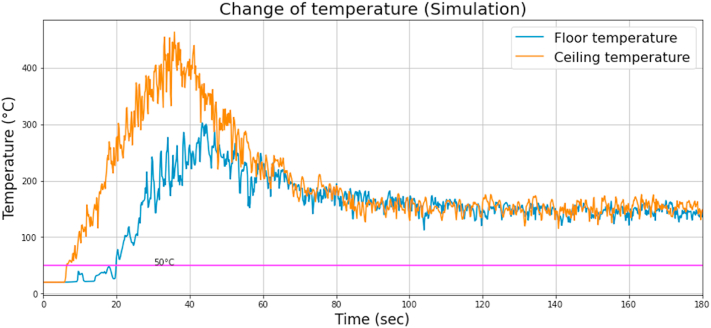
Temperature change during the simulation.

The temperature change is shown in Fig. 6.

**Fig. 7:**
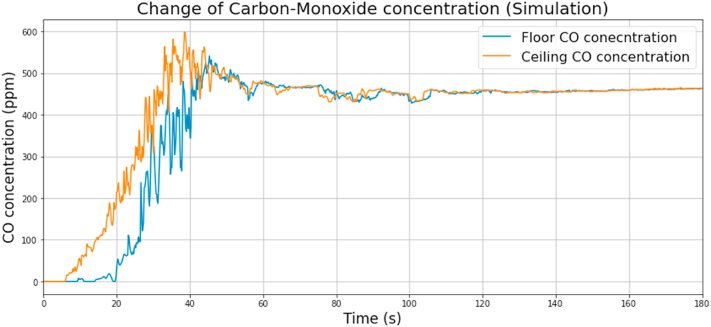
CO concentration change during the simulation.

**Fig. 8:**
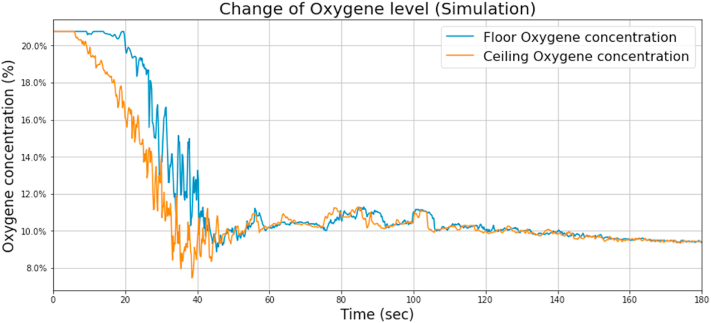
O_2_ concentration change during the simulation.

It can be concluded that both the upper and the lower sensor had a rapid rise in temperature within the first minute. The temperature rose above 50 °C in the case of the lower sensor after 20 s and increased fast shortly after that. This would have decreased the probability of survival of the people in the room. After a short stagnation, the temperature started to decrease because of the lack of oxygen, and heat accumulated in the room. The peak temperature at the ceiling was above 400 °C, which could have damaged the building structure. The CO and the O_2_ concentrations versus time are shown in Figs. 7 and 8, respectively.

Over time, the combustion became more intense with increasing oxygen consumption. As the oxygen level has decreased, combustion became incomplete, increasing CO concentration. The CO concentration also increased rapidly at the sensor below. The CO concentration peak was above 500 ppm, which can cause severe health damage for people.

Based on the analysis of the change of the oxygen concentration, it can be stated that it decreases intensively in the case of both sensors with a slight difference. An oxygen concentration value of around 11% is not enough for combustion with flame.

**Fig. 9:**
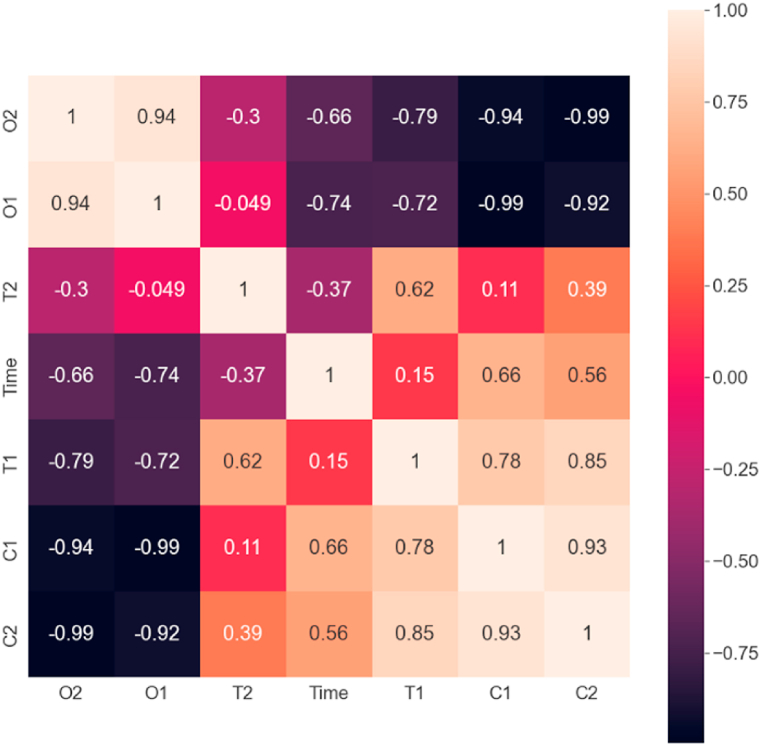
Correlation matrix of the simulation (T1: temperature at the ground, T2: temperature at the ceiling, C1: CO concentration at the ground, C2: CO concentration at the ceiling, O1: O_2_ concentration at the ground, O2: O_2_ concentration at the ceiling).

The correlation analysis result of the simulated data is depicted in Fig. 9 as a normalized correlation matrix. The elements of the correlation matrix were calculated using the Python-SciPy module. The well-known Pearson’s n×n correlation matrix (Eq. (6)) definition was applied to the current $X_{1},X_{2},\ldots ,X_{n}$ multidimensional time-series data to each i,j entry:

(6) $$c_{ij}≔corr\left(X_{i},X_{j}\right)=\frac{cov\left(X_{i},X_{j}\right)}{\sigma_{X_{i}}\sigma_{X_{j}}},if\;\sigma_{X_{i}}\sigma_{X_{j}}>0$$

In the current context, this matrix compares the correlated effect of each variable. In our case, the most significant variable is the oxygen level change, thus the correlation values of oxygen concentration are discussed further. The values of this matrix prove the primary assumptions of simulation results. From the plots, it is visible that as time passes, the oxygen concentration decreases. As expected, the increase in carbon monoxide concentration negatively correlates to the oxygen level. Similarly, as the temperature rises, the oxygen level decreases, and the carbon monoxide level increases as expected. Interestingly, the floor-close temperature does not show a linear relationship with the temperature change close to the ceiling, while indeed has a positive correlation as expected.

### 4.2 1:1 scale fire experiments

**Fig. 10:**
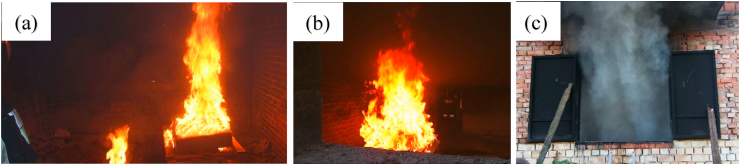
Photos of the fire: (a),(b): The fire, (c): Smoke formation).

The fire was ignited with a diesel fire rod by a firefighter, who then left the room and closed the door. According to the firefighter, the wood shaving quickly caught fire, followed by the foam mattress, and the burning was accompanied by heavy smoke formation (see Fig. 10).

**Fig. 11:**
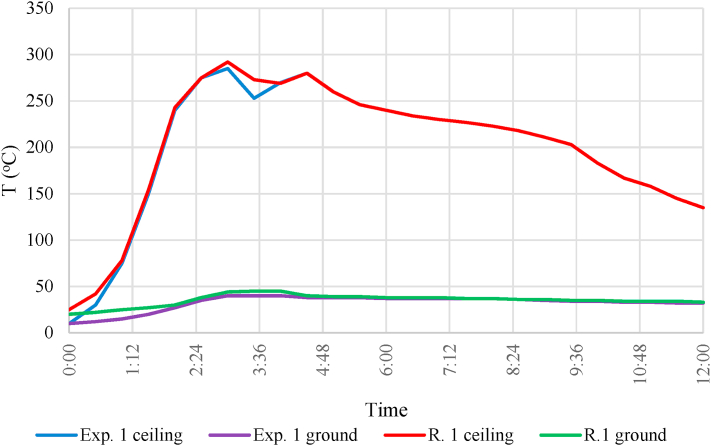
Temperature change during the experiments.

Based on the data from the thermometers, it can be concluded that the temperature increased rapidly during the fire. The temperature reached 282 °C at the height of 2.6 m after 2:57 min. To reduce the fire load on the building structures, the fire extinguishment started to impede any further increment in temperature. Firefighters opened the window and door, and after 10 s, intrusion and firefighting began through the door. The temperature change is illustrated in Fig. 11.

On-site, it was found that smoke generation did not decrease despite the open window. Even the line of sight was reduced to a minimum (from 1.5 m to 0.5 m) due to the smoke and steam generated during firefighting. The temperature measurement was continued after firefighting. The room temperature was still 180 °C at the height of 2.6 m but remained below 50 °C at 0.3 m. The smoke from the room only began to dissipate very slowly. After the fire was extinguished, it took more than 10 min for the vision conditions to improve significantly. The decrease of the temperature to reach below 50 °C measured at the height of 2.6 m took place very slowly. Above the upper plane of the door and window, an enclosed space formed up to the ceiling, where heat and combustion products were trapped. The diagram clearly shows that at 4:30 min, the temperature rises again for a short time, since the water used for firefighting became steam, and the rising cloud of steam condensed the heat and combustion products into the aforementioned upper enclosure. From the fifth minute onwards, a continuous decrease in temperature was observed. The fire unit then ventilated the room with a smoke extraction fan. After complete smoke removal, another hour had to elapse before the temperature of the walls decreased so much that the experiment could be repeated for the second time.

The fire was ignited with a diesel rod by the same firefighter as on the first attempt. After ignition, the firefighter left the room and closed the door. According to the firefighter, the wood shaving was quickly ignited in this experiment as well, and then the foam mattress, the burning was again accompanied by strong smoke formation. According to the data from the thermometers, the temperature increased rapidly during the fire. The temperature reached 292 °C at an altitude of 2.6 m after 3:00 min. To reduce the fire load on the building structures, it was not allowed the temperature to rise further, so the fire fighting started. At the same time, firefighters opened the window and door, and after 10 s, firefighting began through the door. The temperature change is illustrated in Fig. 11.

At the site, it was concluded that the temperature rise was slightly more intense than in the previous experiment. The explanation may be that the walls could not cool down enough compared to the first experiment. The measured temperature was also higher in the case of the sensor placed 0.3 m high, which means a heavier heat load for a person trapped in the room. The accompanying phenomena were the same as described in the previous experiment.

**Fig. 12:**
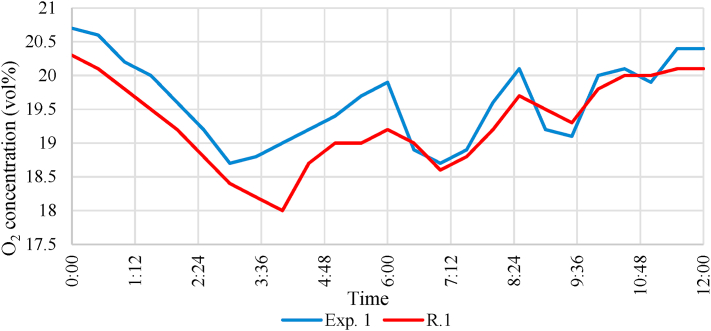
Oxygen (O_2_) concentration change during the experiments.

During the experiments, the volume percentage of O_2_ and CO was also measured with sensors placed at 0.3 m. The oxygen concentration is shown in Fig. 12.

**Fig. 13:**
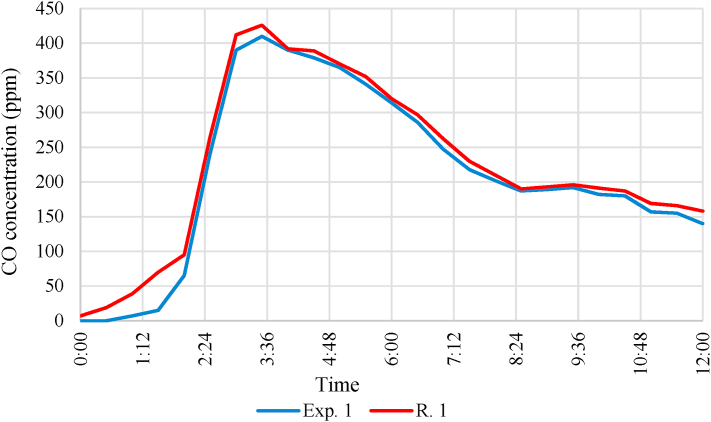
Carbon-monoxide (CO) change during the experiments.

Analysing the data, it can be stated that the O_2_ concentration at the measurement site did not fall below 18 vol% in any of the experiments. In the case of the second experiment, despite the previous ventilation, the initial concentration was 0.4 vol% lower than in the first experiment. During the combustion, the O_2_ concentration decreased. The smallest concentration was measured at the start of the firefighting, after which the concentration increased. It can be concluded that if there was no firefighting intervention, there was still enough oxygen in the room that despite the relatively small amount of combustible material, sufficient heat can be generated for damaging the building structures before the intensity of the combustion would decrease because of lack of O_2,_ as it was observed during simulation. It was also found out that there was still enough oxygen at the height of 0.3 m for a person who might be trapped to survive until the fire units arrived. The CO concentration is shown in Fig. 13.

The data shows that the carbon monoxide (CO) concentration increased intensely. Despite the relatively small amount of combustible material, significant amounts of carbon monoxide were formed during combustion. The concentration of carbon monoxide exceeded the health limit value. Therefore, the trapped person could only survive in this situation with rapid medical intervention.

### 4.3 Comparing simulation and measurement results

**Fig. 14:**
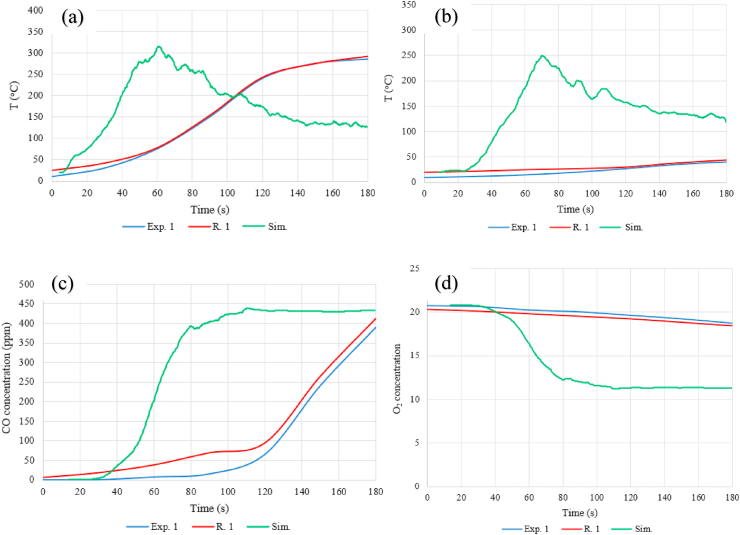
Comparing the simulation and the measurement: (a): Ceiling temperature, (b): Ground temperature, (c): CO concentration, (d): O_2_ concentration).

The comparison of the results of the simulation and the two 1:1 scale experiments is shown in Fig. 14. To eliminate noise, a moving average filter was applied to the simulation results. In the simulation, the rise in the temperature rise and the increase in CO concentration were faster. This observation corresponds to other scientific literature [11]. It can also be concluded that the temperature rise was fast when the temperature reached 300 °C at the ceiling. Therefore, if firefighters did not intervene in the case of the 1:1 scale experiment, the structure would be damaged, and the oxygen concentration would also decrease fast.

It can also be seen that the simulation results differ significantly in terms of temperature at the sensor below. In the case of the experiments, the temperature did not rise above 50 °C near the ground. The CO concentration measured during the experiment was similar to the simulation values. On the other hand, the O_2_ concentration did not decrease below 18 vol% in any experiments. This observation was confirmed by the flame combustion experienced by the firefighters entering the room.

**Fig. 15:**
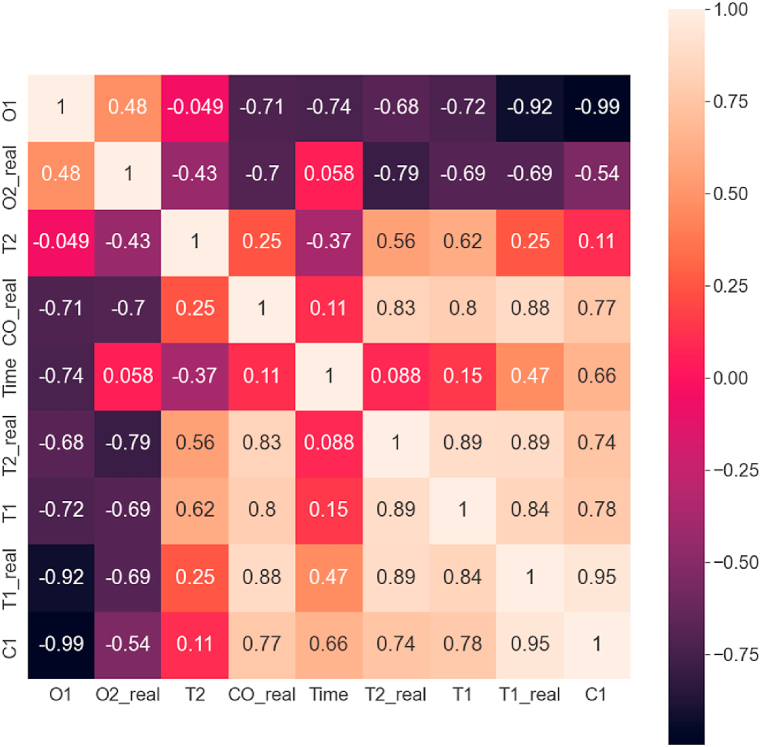
Correlation matrix of the simulation and the experiment (T1: simulated temperature at the ground, T2: simulated temperature at the ceiling, C1: simulated CO concentration at the ground, O1: simulated O_2_ concentration at the ground, T1_real: measured temperature at the ground, T2_real: measured temperature at the ceiling, CO_real: measured CO concentration at the ground, O2_real: measured O_2_ concentration at the ground).

The correlation between real and simulated data is depicted in Fig. 15 as a correlation matrix, with the correlation matrix defined in Eq. (3). The real data (temperature measurements, oxygen level, and carbon monoxide concentration) was approximated with a cubic polynomial to compare with simulated data. Furthermore, the simulated data time was scaled by a factor of four as the simulation run was faster, as it has been investigated by other authors as well. The expected correlation can be observed between the two datasets. Interestingly, the airborne temperature shows a higher correlation with the simulated ground temperature.

## 5 Conclusions

In order to better understand the harmful effects of indoor fires, a computer simulation and two 1:1 scale experiments of an average room fire were performed. Temperature, oxygen (O_2_) concentration, and carbon-monoxide (CO) concentration were monitored in all cases. The main goal of the study was to examine the damage to the structure and the health damage to occupants inside the room. It was found that without firefighting, the temperature can rise fast and cause damage to the building structure. It can also be concluded that after 3 min, the CO concentration can reach such a level that the trapped person could survive only with rapid medical intervention. It was also found out that there was still enough oxygen at the height of 0.3 m for a trapped person to survive until the fire units arrived.

As it is difficult and dangerous to carry out 1:1 scale experiments, a future research task is to improve the simulation model taking into account the results of the fire experiments.
